# Task-dependence in scene perception: Head unrestrained viewing using mobile eye-tracking

**DOI:** 10.1167/jov.20.5.3

**Published:** 2020-05-11

**Authors:** Daniel Backhaus, Ralf Engbert, Lars O. M. Rothkegel, Hans A. Trukenbrod

**Affiliations:** 1 Experimental and Biological Psychology, University of Potsdam, Potsdam, Germany

**Keywords:** scene viewing, real-world scenarios, mobile eye-tracking, task influence, central fixation bias

## Abstract

Real-world scene perception is typically studied in the laboratory using static picture viewing with restrained head position. Consequently, the transfer of results obtained in this paradigm to real-word scenarios has been questioned. The advancement of mobile eye-trackers and the progress in image processing, however, permit a more natural experimental setup that, at the same time, maintains the high experimental control from the standard laboratory setting. We investigated eye movements while participants were standing in front of a projector screen and explored images under four specific task instructions. Eye movements were recorded with a mobile eye-tracking device and raw gaze data were transformed from head-centered into image-centered coordinates. We observed differences between tasks in temporal and spatial eye-movement parameters and found that the bias to fixate images near the center differed between tasks. Our results demonstrate that current mobile eye-tracking technology and a highly controlled design support the study of fine-scaled task dependencies in an experimental setting that permits more natural viewing behavior than the static picture viewing paradigm.

## Introduction

Over the course of the past decades, scene viewing has been used to study the allocation of attention on natural images. In recent years, however, several limitations of the paradigm have been criticized and a paradigmatic shift toward real-world scenarios has been suggested (e.g., Tatler et al., 2011). Here, we propose a different approach that gradually moves from scene viewing toward more natural tasks. This provides a link between the two opposing approaches and helps to understand to which degree eye-movement behavior generalizes across tasks.

In the scene-viewing paradigm, eye movements are recorded in the laboratory from participants looking at an image for a few seconds on a computer screen (Henderson, 2003; Rayner, 2009). Usually, participants get an unspecific instruction to view the image (“free viewing”) or alternatively to memorize the image for a subsequent recall test. In most experiments, images consist of color photographs of the real world selected by the experimenter. As a consequence, within and between experiments, images differ considerably with respect to their low-level features (color, edges), features at more complex levels (shapes, objects, 3D arrangement), and their high-level features (semantic category, action affordances; Malcolm et al., 2016).

One reason why scene viewing has become an intensively used paradigm is that it allows researchers to study eye movements and, hence, the overt allocation of attention on ecologically valid, complex stimuli under highly controlled laboratory conditions. Since the mapping of the eye position to coordinates within an image is straightforward, much research has focused on the question of image-features, influence on eye movements in a bottom-up fashion, that is, independent of the internal state of the observer. Examples of correlations between simple low-level features and fixation positions are local luminance contrast and edge density (Mannan et al., 1997; Reinagel & Zador, 1999; Tatler et al., 2005). But the correlations are not limited to low-level image features. More complex high-level features that correspond to shapes and objects improve predictions substantially (e.g., faces, persons, cars; Cerf et al., 2007; Einhäuser et al., 2008; Judd et al., 2009). The idea of bottom-up selection of fixation locations based on image features led to the development of saliency models (Koch & Ullman, 1985; Itti & Koch, 2001), and a large variety of models has been put forward (e.g., Bruce & Tsotsos, 2009; Kümmerer et al., 2016; Parkhurst et al., 2002). In particular with the development of sophisticated machine-learning algorithms, these models predict fixation locations well when evaluated with a data set obtained under the free viewing instruction (Bylinskii et al., 2016). Beside their influence on fixation locations, both low-level and high-level image features have also been shown to influence fixation durations (Nuthmann, 2017; Tatler et al., 2017).

Already in their anecdotal works, Buswell (1935) and Yarbus (1967) demonstrated that eye-movement patterns depend on the instruction given to the viewer and not just the bottom-up appearance of an image. This top-down influence has often been replicated since (Castelhano et al., 2009; DeAngelus & Pelz, 2009; Mills et al., 2011). Furthermore, in paradigms where participants pursue a specific natural task like preparing a sandwich (Hayhoe et al., 2003) or making a cup of tea (Land et al., 1999), the necessities of motor actions dominate eye-movement behavior. Here, eye movements support task execution by bringing critical information to the foveal region *just-in-time* (Ballard et al., 1997; Land & Tatler, 2009) or as look-ahead fixations on objects needed later during a task (Pelz & Canosa, 2001). Similar conclusions have been made for various other activities like driving (Land & Tatler, 2001), cycling (Vansteenkiste et al., 2014), walking (Matthis et al., 2018; Rothkopf et al., 2007), and ball games (Land & McLeod, 2000; Land & Furneaux, 1997). To align the bottom-up approach with the contradictory findings of top-down control, it is often implicitly assumed that scene viewing without specific instruction provides the means to isolate task-free visual processing. It is a default mode of viewing that can be overridden by the presence of specific tasks. But it is more likely that participants chose a task based on their internal agenda, and researchers are simply unaware of the chosen task in the free viewing condition (Tatler et al., 2011).

In addition, Tatler et al. (2011) criticized several limitations of the scene-viewing paradigm. Participants are seated in front of a computer screen with their head on a chinrest and are asked to minimize head and body movements. Images are presented for a few seconds after a sudden onset on a computer screen, limiting the field of view to the size of the display. The viewpoint is fixed by the photographer and contains compositional biases (Tatler et al., 2005). This is a situation that substantially differs from our experience in daily life, where we are free to move, where scenes emerge slowly (e.g., by opening a door) and our binocular field of view encompasses 200^○^–220^○^ of visual angle (Loschky et al., 2017; Rønne, 1915). As a consequence, visual processing and reconstruction of image content might differ a lot during scene viewing and in real-world tasks as some depth cues (stereo and motion parallax) and motion cues (both egomotion and external motion) are missing in static images. Furthermore, scene viewing utilizes only a portion of the repertoire of eye-movement behaviors needed for other tasks. For example, participants typically make smaller gaze shifts during scene viewing than in everyday activities (Land & Hayhoe, 2001). This is at least in part generated by the restrictions of the task, since saccade amplitudes scale with image size (von Wartburg et al., 2007) and large gaze shifts are usually supported by head movements (Goossens & van Opstal, 1997; Stahl, 1999), but in the classical scene-viewing setup, these head movements are suppressed. Hence, Tatler et al. (2011) suggested to put a stronger emphasis on the study of eye guidance in natural behavior.

Only few studies have directly compared viewing behavior under similar conditions in the real world and in the laboratory. As an exception, ’t Hart et al. (2009) recorded eye movements during free exploration of various indoor and outdoor environments using a mobile eye-tracker. In a second session, the recorded head-centered videos were replayed in the laboratory as a continuous video or randomly chosen frames from the video were presented for 1 s as in the scene-viewing paradigm. Interobserver consistency was highest when observers viewed static images. The result could partially be explained by a bias to fixate near the center, which was strongest in the static image condition as initial fixations are typically directed toward the image center after a sudden onset (cf. Rothkegel et al., 2017; Tatler, 2007). In addition, during free exploration, fixation locations showed a greater vertical variability as participants also looked down on the path while moving forward (cf. ’t Hart & Einhäuser, 2012). Finally, fixations during free exploration were better predicted by fixations from the replay condition than the static image condition, demonstrating that the scene-viewing paradigm has only limited explanatory power for eye movements during free exploration. In a follow-up experiment, Foulsham & Kingstone (2017) demonstrated that keeping the correct order of images in the static image condition changes gaze patterns and improves the predictability of fixation locations during free exploration. But this prediction was no better than just a general bias to fixate near the center independent of image content. In a similar vein, Foulsham et al. (2011) compared eye movements while navigating on a campus with eye movements while watching the head-centered videos. Both conditions showed a strong bias to fixate centrally. However, during walking, gaze was shifted slightly below the horizon, while gaze was shifted slightly above the horizon during watching. Furthermore, while walking participants spent more time looking at the near path, they spent less time on distant objects, and pedestrians were less likely fixated when they approached the observer, in line with the observation that social context modulates the amount of gaze directed toward real people (Laidlaw et al., 2011; Risko et al., 2016).

It is not surprising that eye guidance during scene viewing strongly differs from other natural tasks given the limited overlap of tasks and environments. Even in studies that sought to directly compare laboratory and real-world behavior (Foulsham et al., 2011; Dicks et al., 2010; ’t Hart et al., 2009), several aspects differed between conditions (e.g., size of field of view, task affordances). While scene viewing cannot be thought of as a proxy for eye movements in natural tasks, a paradigmatic shift away from scene viewing might be premature. For several reasons, we advocate for a line of research that makes a smooth transition from the classical scene-viewing paradigm toward more natural tasks. First, the scene-viewing paradigm deals with important aspects of our daily lives as people are constantly engaged in viewing static scenes. Second, the extensive research on scene viewing provides a solid theoretical basis for future research and has led to the development of computational models that predict scanpaths (Engbert et al., 2015; Le Meur & Liu, 2015; Schütt et al., 2017; Schwetlick et al., 2020) and fixation durations (Nuthmann et al., 2010; Tatler et al., 2017). Third, due to the advancement of mobile eye-trackers, it is technically straightforward to address limitations of the paradigm (Tatler et al., 2011), while keeping the benefits of the highly controlled experimental conditions in the laboratory. Fourth, eye guidance in scene viewing is not decoupled from other tasks as some behaviors generalize to other domains. For example, the observation of the central fixation bias (Tatler, 2007), that is, the tendency of viewers to place fixations near the center of an image, has been observed in natural tasks like walking, tea making, and card sorting (’t Hart et al., 2009; Foulsham et al., 2011; Ioannidou et al., 2016). Finally, the scene-viewing paradigm provides a fruitful testbed for theoretical assumptions about eye guidance derived from other paradigms (for example inhibition of return; Rothkegel et al., 2016; Smith & Henderson, 2009) and can advance the development of theories of eye guidance in general.

We suggest to adjust the scene-viewing paradigm step-by-step to deal with its limitations. This approach allows researchers to systematically investigate the influence of individual factors. In this study, we remove some limitations of the paradigm while keeping high overall eye-tracking accuracy. In contrast to the classical scene-viewing paradigm, in our experiment, participants stood in front of a projector screen and viewed images with a specific instruction. Other experimental aspects (e.g., size of field of view, color stimulus material, sudden image onset, possible interactions with the stimulus material) were kept to stay comparable to the classical scene-viewing setup. Eye movements were recorded with a mobile eye-tracker and participants were free to make body and head movements. Note that we did not encourage large-scale head or body movements or force participants to move in front of the screen. But without being explicit, we reduced participants’ restrictions and gave viewers the possibility to move.

The main purpose of our study was to investigate whether established task differences can be reproduced reliably under relaxed viewing conditions. For example, a possible body-posture-related modulation of image-independent fixation tendencies could override task differences that were observed in earlier studies. Thus, the key contribution of this study is to demonstrate the stability of task effects under more natural viewing conditions.

If task effects turn out to be reliable in our paradigm, we expect to find differences in basic eye-movement parameters as in the classical scene-viewing paradigm, for example shorter fixation durations and longer saccade amplitudes for search tasks (Mills et al., 2011; Castelhano et al., 2009). For fixation locations, we expected a more extended range of fixation locations for search tasks (Tatler, 2007). For the central fixation bias, the artificial situation in the laboratory (e.g., sudden image onset; Rothkegel et al., 2017; Tatler et al., 2011) can partly explain the tendency to fixate images near the image center. We expected modulation of the central fixation bias by task since search behavior will typically lead to a broader distribution of fixation locations.

In the following section, we describe our methods, where we outline the processing pipeline to check data quality under this setup and how to convert gaze recorded by a mobile eye-tracker into image coordinates. Next, we report our main results, an early task-independent central fixation bias, and a late task-dependent central fixation bias. We continue with analyses of basic eye-movement parameters such as fixation durations, saccade amplitudes, and distribution of fixation locations across tasks. Finally, we investigate how well fixation locations from one task predict fixation locations from another task in our relaxed setup. We close with a discussion.

## Methods

### Participants

For this study, we used data of 32 students of the University of Potsdam with normal or corrected to normal vision. On average, participants were 22.8 years old (18–36 years) and 31 participants were female. Participants received credit points or a monetary compensation of 10€. To increase compliance with the task, we offered participants an additional incentive of up to 3€  for correctly answering questions after each image (in sum, 60 questions). The work was carried out in accordance with the Declaration of Helsinki. Informed consent was obtained for experimentation from all participants.

### Stimulus presentation, laboratory setup, and procedure

Participants were instructed to look at images while standing in front of a 110-in. projector screen at a viewing distance of 270 cm. Images were projected with a luminance-calibrated video beamer (JVC – DLA-X9500B; frame rate 60 Hz, resolution 1,920×1,080 pixels; Victor Company of Japan, Limited, JVC, Yokohama, Japan). Eye movements were recorded binocularly using the SMI Eye-Tracking Glasses (SMI-ETG 2W; SensoMotoric Instruments, Teltow, Germany) with a sampling rate of 120 Hz. In addition, the scene camera of the Eye-Tracking Glasses recorded the field of view of the participant with a resolution of 960×720 pixels (60^○^× 46^○^ of visual angle) at 30 Hz.

All images were presented with a resolution of 1,668×828 pixels at the center of the screen. Images were embedded in a gray frame with QR-markers (126 × 126 pixels; cf. Figure 2) and covered 40.6^○^ of visual angle in the horizontal and 20.1^○^ in the vertical dimension. Images were colored scene photographs taken by the authors; every single image contained zero to 10 humans and zero to 10 animals. We used 27 images with people and animals, one image with only animals, one image with only people, and one image with neither people nor animals. Furthermore, images were selected by having an overall sharpness, were taken in different countries, and did not contain prominent text. Each of the 30 images could appear in every condition and was presented in two conditions to every single participant.

The experiment consisted of four blocks. In each block, participants viewed images under one of four instructions. Under two instructions, participants had to count the number of people (Count People) or count the number of animals in an image (Count Animals). Under the two remaining instructions, participants had to guess the time of day when an image was taken (Guess Time) and guess the country in which an image was taken (Guess Country). We expected the count instructions to resemble search tasks, since the entire image had to be thoroughly examined to give a correct answer, while the guess instructions were thought to resemble the free viewing instruction but with a stronger focus on one aspect of the image for all participants. In each block, we presented 15 images for 8 s. While the order of instructions was counterbalanced across participants, each image was randomly assigned to two of the four instructions.

**Figure 1.**
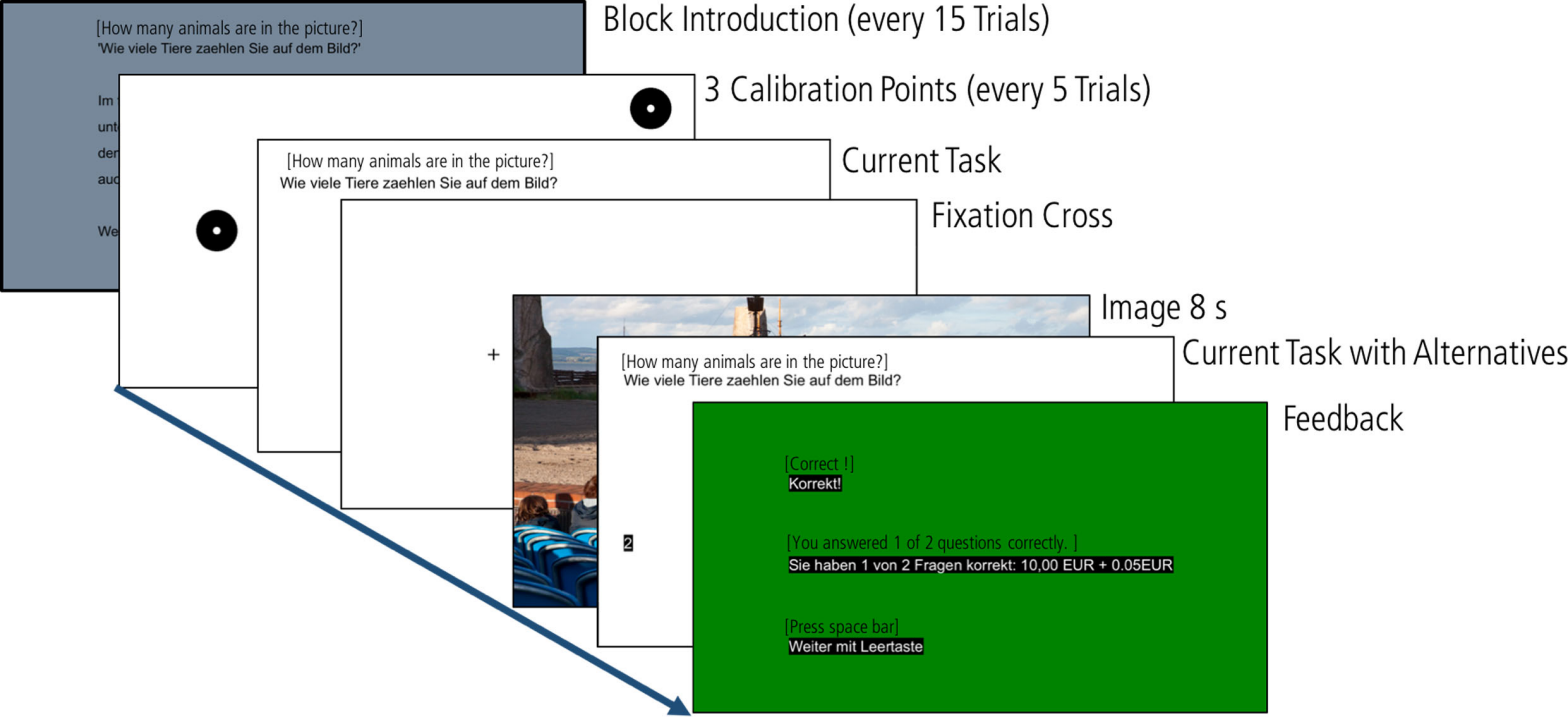
Sequence of events in the scene-viewing experiment.

At the beginning of each block we presented a detailed instruction for the upcoming task, followed by a three-point calibration (Figure 1). Individual trials began with a 1 s reminder of the instruction, followed by a black fixation cross (0.73^○^ × 0.73^○^) presented on a white background for 3 s. Participants were instructed to fixate the fixation cross until the image appeared. Fixation crosses appeared on a grid of 15 fixed positions: three vertical positions (25%, 50%, and 75% of the projector screen's vertical size) and five horizontal positions (20%, 35%, 50%, 65%, and 80% of the projector screen's horizontal size). Afterward, participants were free to explore the image for 8 s. At the end of a trial, participants had to answer orally a multiple-choice question with three alternatives presented on the screen. We gave immediate feedback, and each correct answer was rewarded with 0.05€. The instructor pressed a button to continue with the next trial, which started with a brief reminder of the instruction. The eyes were calibrated at the beginning of each block and after every fifth image. In addition, instructors could force a new calibration after a trial if fixations deviated more than ∼1^○^ from the fixation cross during the initial fixation check.

### Raw data processing

#### Transformation

**Figure 2.**
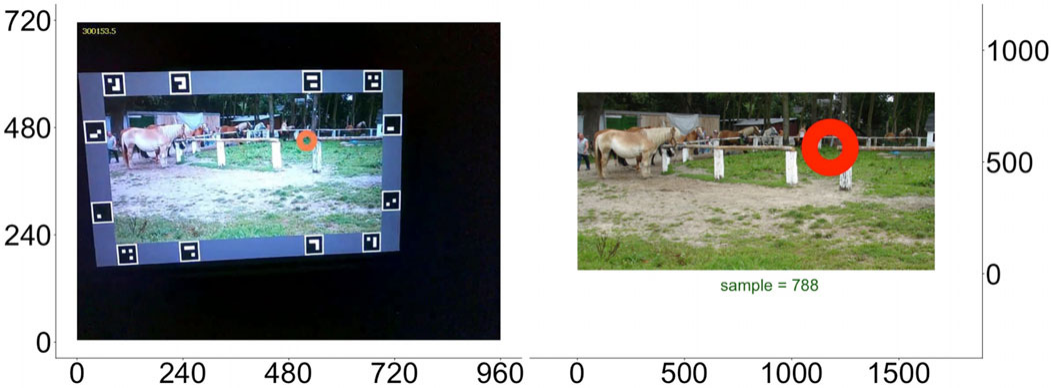
Transformation of scene-camera coordinates (subpixel level) into image coordinates in pixels. Left panel: Frame taken by SMI ETG-120Hz scene camera with measured fixation location (circle). Right panel: The same frame and fixation in image coordinates.

The experimentally measured eye positions were given in coordinates of the scene camera of the mobile eye-tracker. Thus, raw data subpixel (1/100 pixel) values had to be transformed into coordinates of the presented image (Figure 2). To achieve this, we used a projective transformation provided by the computer vision toolbox in the MATLAB programming language (MATLAB 2015b; The MathWorks, Natick, MA, USA). The required locations of image corners were extracted from the scene-camera output frame by frame, using 12 unique QR-markers, which were presented around the images. Automatic QR-marker detection and detection of image corners were done with the Offline Surface Tracker module of the Pupil Labs software Pupil Player version 1.7.42 (Kassner et al., 2014). To synchronize the time of both devices, we sent UDP-messages from the presentation computer to the recording unit of the eye-tracker. As a result of this calculation, we worked with three trajectories in image coordinates: two monocular data streams and one binocular data stream. First, saccade detection was performed with both monocular eye-data streams (see next section). Second, we calculated mean fixation positions based on the binocular eye-data stream (note that the binocular data are not the simple mean of both monocular trajectories). Pilot analyses of the fixation positions indicated higher reliability of the binocular position estimate compared to averaging of monocular positions.

#### Saccade detection

For saccade detection, we applied a velocity-based algorithm (Engbert & Kliegl, 2003; Engbert & Mergenthaler, 2006). The algorithm marks all parts of an eye trajectory as a saccade that have a minimum amplitude of 0.5^○^ and exceed a velocity threshold for at least three successive data samples (16.7 ms). The velocity threshold is computed as a multiple λ of the median-based standard deviation of the eye trajectories velocity during a trial. We carried out a systematic analysis with varying threshold multipliers λ to identify detection parameters for obtaining robust results (Engbert et al., 2016). Here, we computed the velocity threshold with a multiplier λ = 8. We first analyzed both monocular eye trajectories to identify potential saccades and kept all binocular events.

Following Hessels et al. (2018), it is important to clearly define what a fixation means in the context of a specific analysis. In the current work, fixations refer to moments of relative stability on an image, regardless of eye-in-head and body movements. Fixations were computed as the epoch between two subsequent saccades. The binocular eye-data stream provided from the recording unit was transformed and used to calculate the mean fixation position.

### Data quality

#### Raw data quality

In total, we recruited 42 participants to get our planned 32 participants. Five participants had to be replaced as the experimenter was not able to calibrate them reliably (these participants did not finish the experiment). Another five participants had to be replaced since at least a fifth of their data was missing due to blinks and low data quality (see next paragraph).

To ensure high data quality, we marked blinks and epochs with high noise in the eye trajectories. For the detection of blinks, we made use of the blink detection provided by the SMI-ETG 2W. All fixations and saccades that contained a blink as well as all fixations and saccades with a blink during the preceding or succeeding event were removed from further analyses. Several other criteria were applied to detect unreliable events. First, we detected instable fixations (e.g., due to a strong jitter in the signal of the eye trajectory) by calculating the mean 2D standard deviation of the eye trajectory of all fixations. All fixations that contained epochs that exceeded the 2D standard deviation by a factor of 15 were removed from further analyses. Second, as saccades are stereotyped and ballistic movements, all saccades with a duration of more than 250 ms (30 samples) were removed. These saccades would be expected to have amplitudes, which go far beyond the dimensions of the projector screen; further, we removed all saccades with amplitudes greater than or equal to 25^○^. Third, we removed fixations located outside the image coordinates and fixations with a duration of less than 25 ms as well as with durations of more than 1,000 ms. As a final criterion, we calculated the absolute deviation of participants’ eye positions from the initial fixation cross. We computed the median deviation of the last 200 ms before the appearance of an image. Since we were not able to cancel the next trial and to immediately recalibrate with our setup, we removed trials with an absolute deviation greater than 2^○^. Overall, 40,182 fixations (∼81% of 49,371) and 37,726 saccades (∼80% of 47,425) remained for further analyses.

#### Main sequence of saccade amplitude and peak velocity

**Figure 3.**
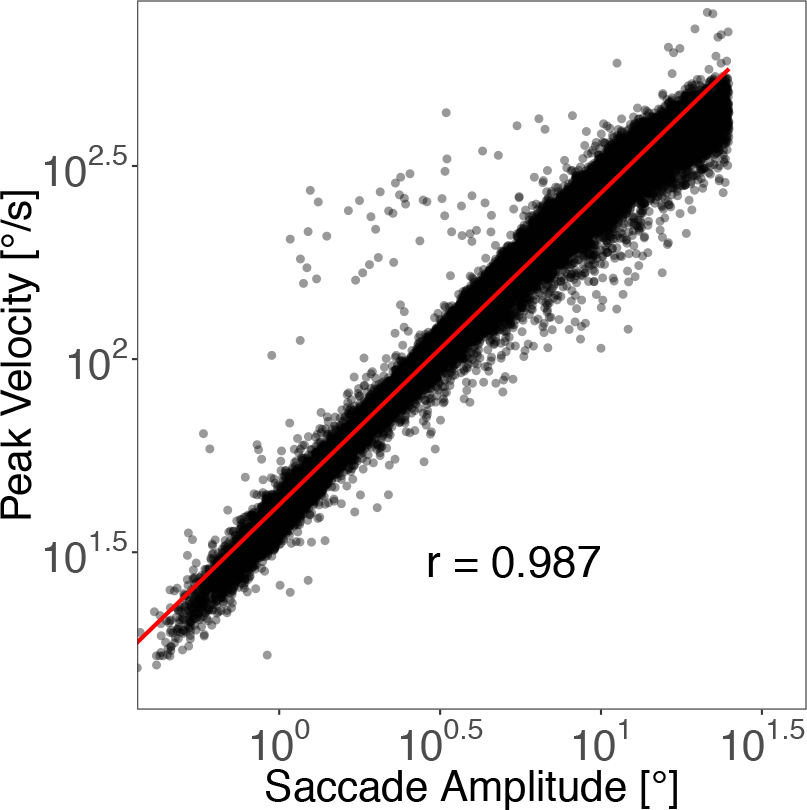
Main sequence. Double-logarithmic representation of saccade amplitude and saccade peak velocity.

Since saccades are stereotyped and ballistic movements, there is a high correlation between a saccade's amplitude and its peak velocity. We investigated this relationship by computing the main sequence, that is, the double-logarithmic linear relation between saccade amplitude and peak velocity (Bahill et al., 1975). The 37,726 saccades in our data set range from about 0.5^○^ to about 25^○^ of visual angle, due to our exclusion criteria (Figure 3). There is a strong linear relation in the main sequence with a very high correlation, *r* = .987. Hence, the detected saccades behaved as expected and were used for further analyses.

#### Head and body movements

**Figure 4.**
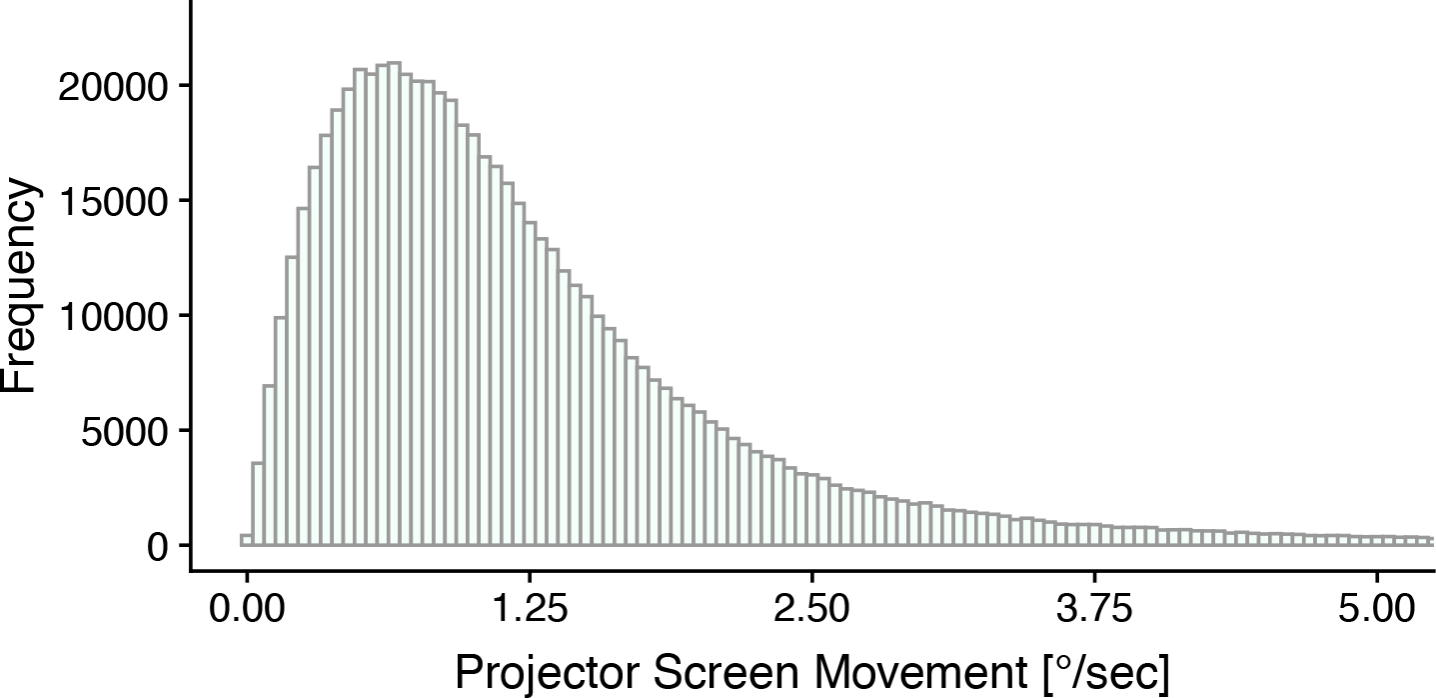
Projector screen movement. As an approximation of head movements, the projector screen movement is measured by tracking the position of QR-markers in the scene-camera video.

We realized a more natural body posture by recording without a chinrest and thereby enabling for small body and head movements in front of a projector screen. Even so, we did not expect large-scale head or body movements, as we did not encourage gestures or movements explicitly in our tasks (Epelboim et al., 1995). For an approximating measure of participants’ movements in front of the screen, we made use of the QR-markers presented around the images. By tracking the marker positions in the scene-camera video, we receive a measure of participants’ head position and angle relative to the projector screen. Figure 4 shows the distribution of the projector screen movements as an approximation for head and body movements. The distribution has a peak at around 1^○^/s and only few samples with velocities ≥2.5^○^/s. Thus, the majority of values do not exceed the velocities of fixational eye movements.

#### Accuracy of the eye position

**Figure 5.**
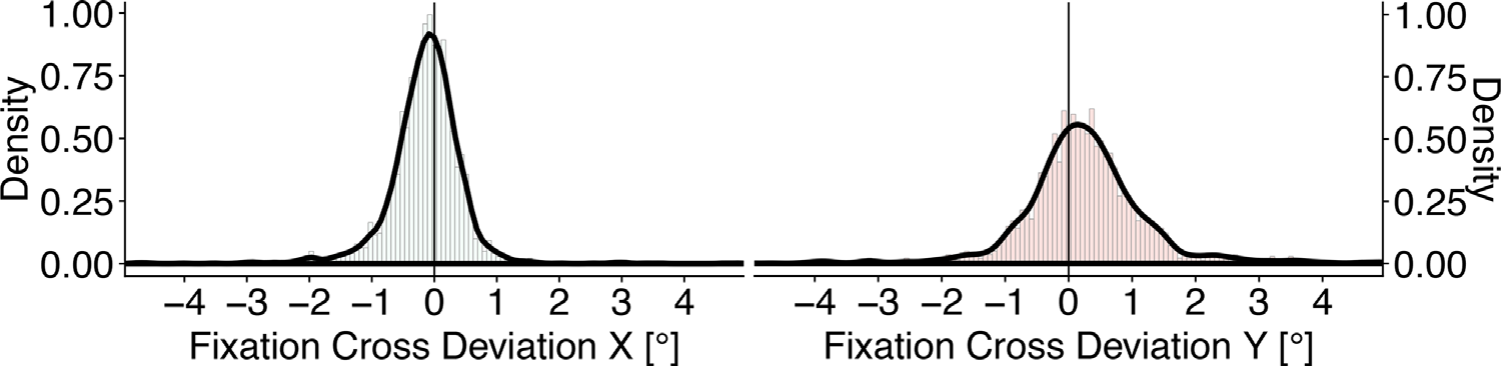
Median horizontal and vertical deviation of participants’ gaze position from the initial fixation cross in the left and right panels, respectively.

Finally, at least two error sources contribute to the accuracy of the measured eye position in our setup: measurement error generated by the eye-tracking device and the calibration procedure as well as error generated by the transformation of the eye position from scene-camera coordinates into image coordinates. To estimate the overall spatial accuracy of our setup, we calculated the deviation of participants’ gaze positions from the initial fixation cross. For each fixation check, we computed the median difference of the gaze position minus the position of the fixation cross for the last 200 ms (24 samples) of the fixation check. Figure 5 shows the distributions of deviations from the initial fixation cross in the horizontal (left panel) and vertical (right panel) dimension. Horizontal deviations are mostly within 1^○^ of visual angle (91.04%) with a small leftward shift. The distribution of vertical deviations is slightly broader (76.65% within 1^○^ of visual angle) with a small upward shift. Thus, overall accuracy of our experimental setup is good but, as expected, somewhat weaker than in scene-viewing experiments using high-resolution eye-trackers. Note, Figure 5 contains trials that were subsequently excluded from further analysis since their absolute deviation exceeded 2^○^.

### Analyses

Beside the analysis of fixation durations and saccade amplitudes, we used three further metrics to describe the eye-movement behavior in our experiment. First, to quantify the central fixation bias (Tatler, 2007), we computed the distance to image center over time (Rothkegel et al., 2017). Second, as an estimate for the overall dispersion of fixation locations on an image, we computed the informational entropy (Shannon & Weaver, 1963). Third, we evaluated how well fixation positions can be predicted by a distribution of fixation locations (Schütt et al., 2019), for example, computed from a different set of fixation locations or obtained as the prediction of a computational model. We computed linear mixed-effect models (LMMs) for each dependent variable using the lme4 package (Bates et al., 2015) in R (R Core Team, 2019). If the dependent variable deviated remarkably from a normal distribution, we performed a log-transform. For the statistical model of the empirical data, we used the task as a fixed factor and specified custom contrasts (Schad et al., 2018). First, we compared the two Guess tasks against the two Count tasks. Second, we tested the Count Animals against the Count People condition. The third contrast coded the difference of the Guess Time and the Guess Country condition. The models were fitted by maximum likelihood estimation. For the random effect structure, we ran a model selection further described in Supplementary Appendix S1. Following Baayen et al. (2008), we interpret all |*t*| > 2 as significant fixed effects.

#### Central fixation bias

The central fixation bias (Tatler, 2007) refers to the tendency of participants to fixate near the image center. The bias is strongest initially during a trial and reaches an asymptotic level after a few seconds. To describe this tendency, we computed the mean Euclidian distance Δ(*t*) of the eyes to the image center over time (Rothkegel et al., 2017),

(1) $$\Delta \left(t\right)=\frac{1}{m*n}\sum_{j=1}^{m}\sum_{k=1}^{n}\left|\right|x_{jk}\left(t\right)-x^{'}\left|\right|,$$

where *x*_*jk*_ refers to the gaze coordinates of a participant *j* on image *k* at time *t* and *x*^′^ refers to the coordinates of the image center. If fixations were uniformly placed on an image, a value of 12^○^ would be expected, which is the average distance of every pixel to the image center. Note, here we chose to compute the distance to image center Δ(*t*) for specific time intervals *t*: 0 to 400 ms, 400 to 800 ms, 800 to 1,200 ms, and 1,200 to 8,000 ms. These time intervals were chosen because previous work has shown that the first 400 ms of a scanpath show more reflexive saccades in response to the image onset, and after 400 ms, content- or goal-driven saccades are executed (Rothkegel et al., 2017). Thus, these later saccades are more likely to be influenced by the specific viewing task.

#### Entropy

We use information entropy (Shannon & Weaver, 1963) to characterize the degree of uniformity of a distribution of fixation locations. We calculate the entropy by first estimating the density of a distribution of fixation locations on a 128 × 128 grid. The density is computed in R using the spatstat package (Baddeley & Turner, 2005) with an optimal bandwidth for each distribution of fixation locations (bw.scott). After transforming the density into a probability measure (integral sums to 1), the entropy *S* is measured in bits and computed as

(2) $$S=-\sum_{i=1}^{n}p_{i}\log_{2}p_{i}\;,$$

where each cell *i* of the grid is evaluated. In our analysis, an entropy of 14 bits (*n* = 128 × 128 = 2^14^) represents the maximum degree of uniformity, that is, the same probability of observing a fixation in each cell; a value of 0 indicates that all fixations are located in only one cell of the grid.

#### Predictability

Finally, we estimated the negative cross-entropy of two fixation densities to quantify to what degree a set of fixation locations is predicted by a given probability distribution. The metric can be used to investigate how well an empirically observed fixation density (e.g., from a set of fixations recorded from other participants) or the fixation density generated by a computational model (e.g., a saliency model) predicts a set of fixation locations (Schütt et al., 2019). The negative cross-entropy *H*(*p*_2_; *p*_1_) of a set of *n* fixations can be approximated by

(3) $$H\left(p_{2};p_{1}\right)\approx -\frac{1}{n}\sum_{i=1}^{n}\log_{2}\left({\hat{p}}_{1}\left(f_{2}^{\left(i\right)}\right)\right)\;,$$

where ${\hat{p}}_{1}$ refers to a kernel-density estimate of the fixation density *p*_1_, which is evaluated at the fixation locations $f_{2}^{\left(i\right)}$ of a second fixation density *p*_2_. The log-likelihood measure approximates how well *p*_1_ approximates *p*_2_ irrespective of the entropy *p*_2_. We implemented the negative cross-entropy with a leave-one-subject-out cross-validation. For each participant on each image and each task, we computed a separate kernel-density estimate ${\hat{p}}_{1}$ by using only the fixations of all other participants viewing the same image under the same instruction.

In our analyses, we computed fixation densities ${\hat{p}}_{1}$ on the same 128 × 128 grid used for the entropy computations. All empirical densities (from sets of fixation locations) were computed in R using the spatstat package (Baddeley & Turner, 2005) with a bandwidth determined by Scott's rule for each distribution (bw.scott). In addition, we used fixation densities predicted by a state-of-the-art saliency model (Kümmerer et al., 2016). All density distributions were converted into probability distributions (intergral sums to 1) before computing the negative cross-entropy *H*(*p*_2_; *p*_1_). A value of $0\;\frac{\mathrm{bit}}{\text{fix}}$ demonstrates perfect predictability. A value of $-14\;\frac{\mathrm{bit}}{\text{fix}}\;\mathrm{since}\;128\times 128=2^{14}$ is expected for a uniform probability distribution, where all locations in the probability distribution are equally likely to be fixated. In the Results section, we report Δ log-likelihoods that indicate the gain in predictability of the negative cross-entropy relative to a uniform distribution.

## Results

In the Methods section, we ensured that the workflow necessary to measure eye movements in a relaxed version of the scene-viewing paradigm provides data quality comparable to the laboratory setup. Next, we wanted to see if it is possible to replicate task differences under this setup. As the most commonly used eye-movement parameters, we first analyzed fixation durations and saccade amplitudes. Next, we examined the distributions of fixation locations to quantify systematic differences in target selection between tasks. We compared the strength of the central fixation bias in the four tasks. A direct within-subject comparison of the central fixation bias on the same stimulus material has not been reported before. We computed the entropy to quantify the overall dispersion of fixation locations on an image, computed a log-likelihood to see how well fixations can be predicted across tasks, and compared fixation locations in the four tasks with the predictions of a saliency model.

In our Results section, we report linear mixed-effect model (LMM) analyses. Moreover, we used post hoc multiple comparisons to further investigate differences between tasks. All reported *p* values in the multiple comparisons were adjusted according to Tukey. A summary of all investigated eye-movement parameters can be found in Table 1.

**Table 1.** Mean values of eye-movement parameters under the four task instructions. The central fixation bias (CFB) is reported as the average distance Δ(*t*) to the image center during specific time intervals *t*.

	Count People	Count Animals	Guess Country	Guess Time
Fixation duration [ms]	249	233	244	248
Saccade amplitude [^○^]	6.27	6.45	6.76	6.83
CFB[^○^]: 0 to 400 ms	5.809	5.573	5.730	5.596
CFB[^○^]: 400 to 800 ms	7.678	7.203	6.740	6.420
CFB[^○^]: 800 to 1,200 ms	9.672	9.552	8.551	8.482
CFB[^○^]: 1,200 to 8,000 ms	10.351	10.899	9.821	9.688
Entropy [bit]	13.051	13.476	13.327	13.394
Predictability [bit/fix]	1.187	0.745	0.936	0.830
DeepGaze2 [bit/fix]	0.434	−0.101	0.726	0.562

### Fixation durations

**Figure 6.**
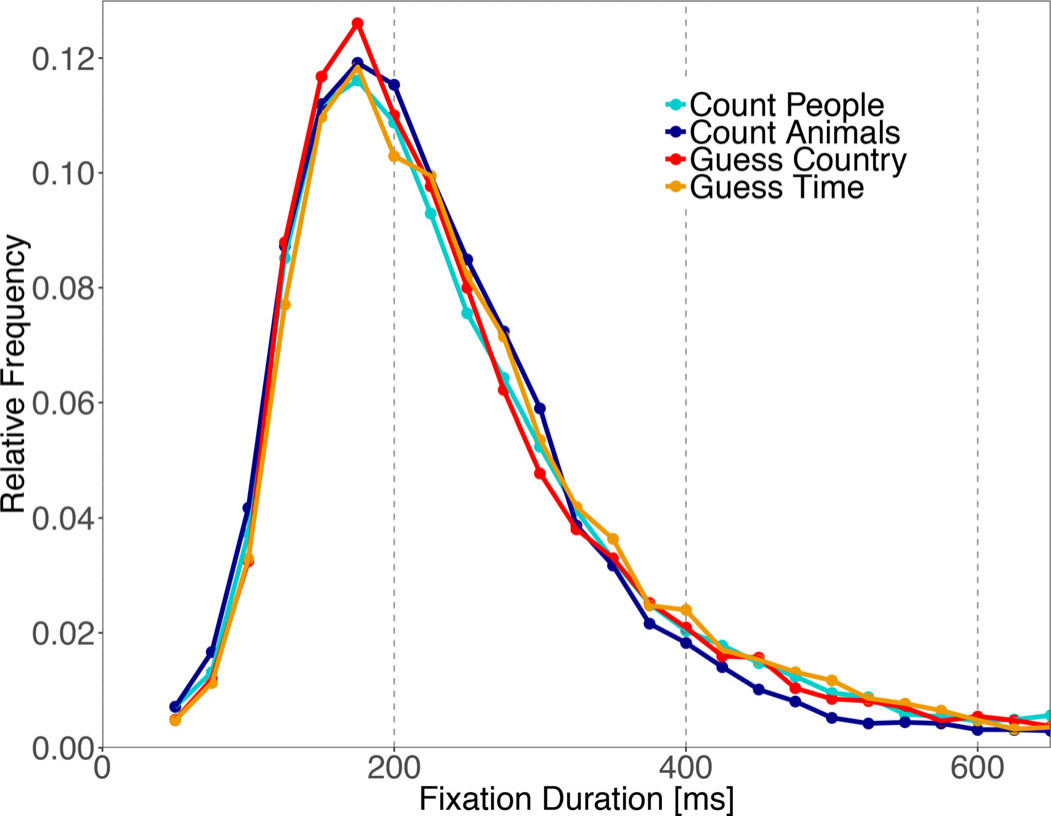
Fixation duration distributions. The figure shows relative frequencies of fixation durations in the four tasks. Fixation durations were binned in steps of 25 ms.

Distributions of fixation durations for the four different tasks are plotted in Figure 6. All distributions show the characteristic form typically observed for eye movements in scene viewing. The distributions in our tasks peak at around 200 ms and show a long tail with fixation durations above 400 ms. A LMM (see Methods section; Bates et al., 2015) revealed significant fixed effects of task (Table 2). All of our comparisons, specified by our three contrasts, show significant differences. To ensure the normal distribution of model residuals, fixation durations were log-transformed. Fixation durations were shortest in the Count Animals condition (233 ms) and post hoc multiple comparisons revealed that fixation durations in this task differed significantly from all other tasks (all *p* ≤ 0.05; Table 3). The effect seem to be primarily driven by a reduction of long fixation durations in the range between 350 and 550 ms (blue line in Figure 6). There were no reliable differences in fixation durations between Count People and the Guess conditions (all *p* > 0.5; Count People: 249 ms, Guess Country: 244 ms, Guess Time: 248 ms). Replicating the results from the linear mixed-effect model, the Guess conditions also differed significantly in the post hoc multiple comparisons analysis (*p* < 0.001).

**Table 2.** Fixed effects of linear mixed − effect model (LMM): Fixation durations (log-transformed) for our contrasts. *Note*: |*t*| > 2 are interpreted as significant effects.

	β	*SE*	*t*
Guess - Count	0.02	0.01	2.16
Count Animals - CountPeople	−0.05	0.01	−4.80
GuessTime - GuessCountry	0.03	0.01	3.62

**Table 3.** Multiple comparisons of fixation durations (log-transformed) for all tasks. Adjusted *p* values reported (Tukey). Levels of significance: ***p < 0.001, **p < 0.01, *p < 0.05.

Post hoc comparison	Estimate	*SE*	*z* value	Pr(>|z|)
Count Animals - Count People	−0.054	0.0112	−4.796	<0.001***
Guess Country - Count People	−0.017	0.0129	−1.278	0.564
Guess Time - Count People	0.010	0.0129	0.800	0.848
Guess Country - Count Animals	0.037	0.0129	2.889	0.019*
Guess Time - Count Animals	0.064	0.0129	4.983	<0.001***
Guess Time - Guess Country	0.027	0.0074	3.620	0.002**

**Table 4.** Fixed effects of linear mixed − effect model (LMM): Saccade amplitudes (log-transformed) for our contrasts. *Note*: |*t*| > 2 are interpreted as significant effects.

	β	*SE*	*t*
Guess - Count	0.10	0.03	3.95
CountAnimals - CountPeople	0.06	0.04	1.56
GuessTime - GuessCountry	0.01	0.01	0.43

### Saccade amplitudes

**Figure 7.**
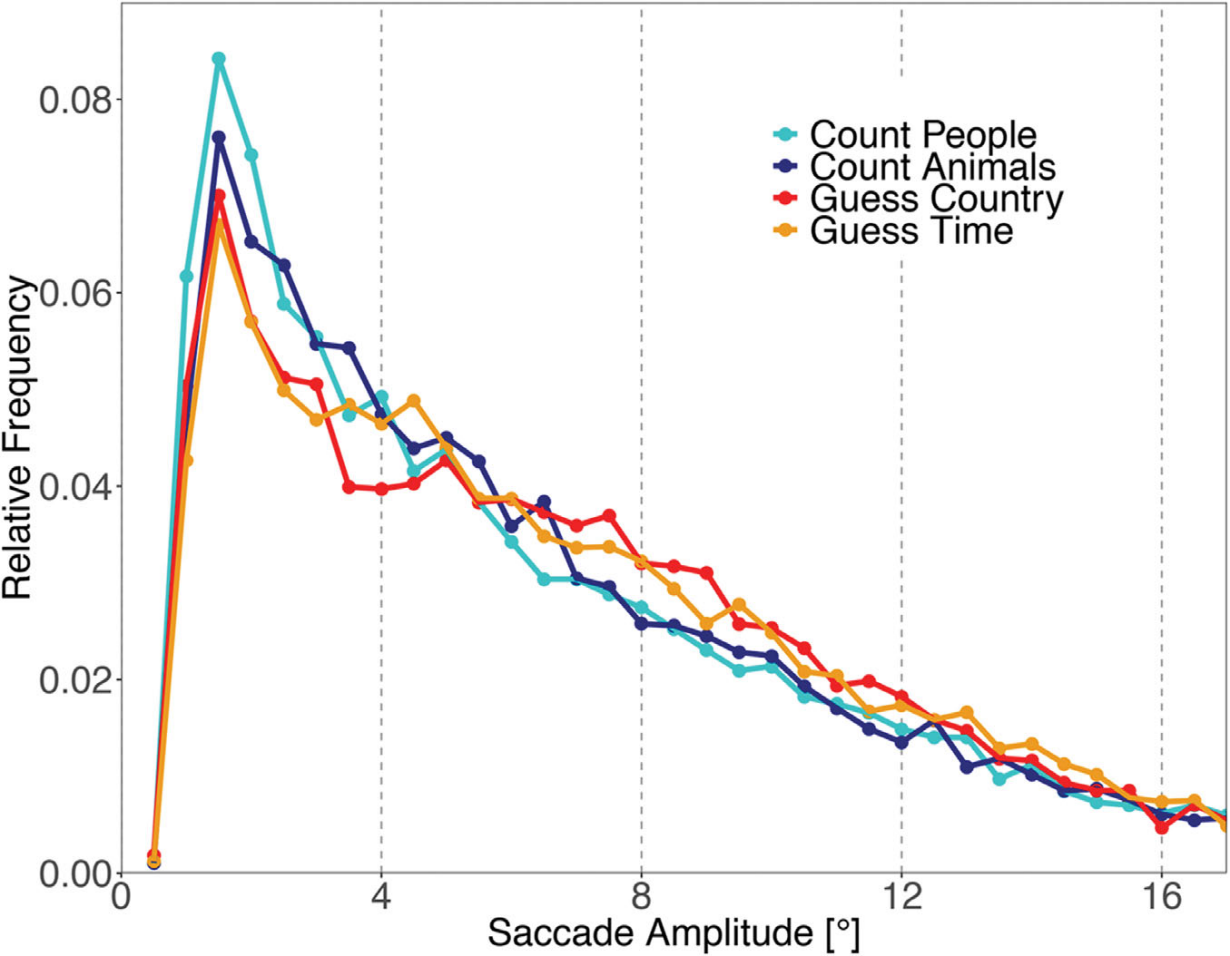
Distribution of saccade amplitudes. The figure shows relative frequencies of saccade amplitudes in the four tasks. Saccade amplitudes were binned in steps of 0.5^○^.

Relative frequencies of saccade amplitudes for the four tasks are shown in Figure 7. In line with previous scene-viewing experiments, saccade amplitude distributions show a peak between 2^○^ and 3^○^ with a substantial proportion of larger saccades. A LMM revealed a significant difference across the Guess and Count tasks for saccade amplitudes (log-transformed since saccade amplitudes deviated considerably from a normal distribution). Both within Guess and within Count conditions were not significant (Table 4). Post hoc multiple comparisons revealed significant differences between Count People and Guess conditions (all *p* < 0.001; Table 5). Saccade amplitudes in the Guess Country (6.76^○^) and Guess Time condition (6.83^○^) were longer on average than saccade amplitudes in the Count People (6.27^○^) condition. There were no other significant differences (all *p* > 0.09).

**Table 5.** Multiple comparisons of saccade amplitudes (log-transformed) for all tasks. Adjusted *p* values reported (Tukey). Levels of significance: ***p < 0.001, **p < 0.01, *p < 0.05.

Post hoc comparison	Estimate	*SE*	*z* value	Pr(>|*z*|)
Count Animals - Count People	0.059	0.038	1.560	0.380
Guess Country - Count People	0.127	0.032	3.923	<0.001***
Guess Time - Count People	0.133	0.032	4.100	<0.001***
Guess Country - Count Animals	0.068	0.032	2.104	0.138
Guess Time - Count Animals	0.074	0.032	2.278	0.093
Guess Time - Guess Country	0.006	0.013	0.430	0.971

### Central fixation bias

**Figure 8.**
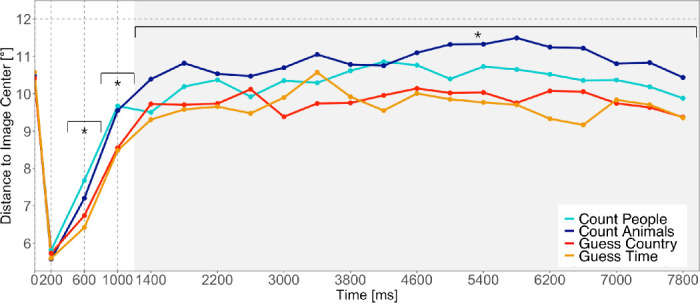
Temporal evolution of the central fixation bias measured as the average distance to image center. Each line corresponds to one of the four instructions. The horizontal line provides the expected distance to center, if fixations were uniformly placed on an image. Level of significance: *p < 0.05.

The central fixation bias (CFB) is a systematic tendency of observers to fixate images, presented on a computer screen, near their center (Tatler, 2007) and is strongest during initial fixations (Rothkegel et al., 2017; Tatler, 2007; ’t Hart et al., 2009). We measured the CFB as the distance to the image center (Equation 1) and found a strong initial CFB in all conditions (Figure 8). Before the first saccade, participants’ gaze positions were located on the initial fixation cross. The earliest subsequent fixations of the exploration were on average closest to the image center. All later fixations were less centered and the average distance to image center reached an asymptotic level after 1,000 to 2,000 ms. We computed the average distance of all image coordinates from the image center. A distance to image center of 12^○^ would be expected if fixations were uniformly placed on the image.

We compared the distance to image center in the four tasks with LMMs for specific time intervals. There was no significant fixed effect of task during the earliest fixations (0 to 400 ms; Table 6), but we observed differences between tasks for all later time intervals: for fixations in between 400 and 800 ms, we found that Guess and Count conditions as well as Count People and Count Animals conditions differed significantly. Fixations in between 800 and 1,200 ms differed significantly between Guess and Count conditions, but we could not find significant differences in between Guess and Count conditions. For later fixations (1,200 to 8,000 ms), all fixed effects show significant differences. Post hoc multiple comparisons revealed no significant differences between tasks for the earliest fixations (0 to 400 ms) (all *p* >.3; Table 7).

**Table 6.** Fixed effects of linear mixed − effect models (LMM): Distance to image center across tasks for different time intervals for our contrasts. *Note*: |*t*| > 2 are interpreted as significant effects.

Time interval	β	*SE*	*t*
*0 to 400 ms*			
Guess - Count	−0.11	0.15	−0.70
Count Animals - CountPeople	−0.20	0.21	−0.96
GuessTime - GuessCountry	−0.26	0.22	−1.21
*400 to 800 ms*			
Guess - Count	−0.80	0.14	−5.92
Count Animals - CountPeople	−0.47	0.19	−2.52
GuessTime - GuessCountry	−0.23	0.20	−1.15
*800 to 1,200 ms*			
Guess - Count	−1.02	0.18	−5.71
Count Animals - CountPeople	−0.01	0.25	0.05
GuessTime - GuessCountry	−0.22	0.26	0.82
*1,200 to 8,000 ms*			
Guess - Count	−0.89	0.05	−16.42
Count Animals - CountPeople	0.55	0.08	7.43
GuessTime - GuessCountry	−0.24	0.08	−3.10

**Table 7.** Multiple comparisons of distance to image center across tasks for different time intervals. Adjusted *p* values reported (Tukey). Levels of significance: ***p < 0.001, **p < 0.01, *p < 0.05.

Post hoc comparison	Estimate	*SE*	*z* value	Pr(>|*z*|)
*Fixations 0 to 400 ms*				
Count Animals - Count People	−0.199	0.206	−0.963	0.771
Guess Country - Count People	−0.073	0.214	−0.340	0.986
Guess Time - Count People	−0.338	0.211	−1.598	0.380
Guess Country - Count Animals	0.126	0.214	0.588	0.936
Guess Time - Count Animals	−0.139	0.212	−0.655	0.914
Guess Time - Guess Country	−0.265	0.220	−1.205	0.623
*Fixations 400 to 800 ms*				
Count Animals - Count People	−0.468	0.186	−2.518	0.057
Guess Country - Count People	−0.923	0.190	−4.853	<0.001***
Guess Time - Count People	−1.151	0.192	−6.007	<0.001***
Guess Country - Count Animals	−0.455	0.192	−2.370	0.083
Guess Time - Count Animals	−0.682	0.195	−3.507	0.003**
Guess Time - Guess Country	−0.227	0.198	−1.147	0.660
*Fixations 800 to 1,200 ms*				
Count Animals - Count People	0.013	0.246	0.054	1.000
Guess Country - Count People	−1.125	0.253	−4.452	<0.001***
Guess Time - Count People	−0.908	0.260	−3.491	0.003**
Guess Country - Count Animals	−1.138	0.248	−4.595	<0.001***
Guess Time - Count Animals	−0.922	0.255	−3.613	0.002**
Guess Time - Guess Country	0.216	0.263	0.824	0.843
*Fixations 1,200 to 8,000 ms*				
Count Animals - Count People	0.551	0.075	7.343	<0.001***
Guess Country - Count People	−0.496	0.078	−6.345	<0.001***
Guess Time - Count People	−0.741	0.077	−9.588	<0.001***
Guess Country - Count Animals	−1.048	0.077	−13.672	<0.001***
Guess Time - Count Animals	−1.293	0.076	−17.029	<0.001***
Guess Time - Guess Country	−0.245	0.079	−3.104	0.010*

On the following time interval (400 to 800 ms), fixations in the Count People condition were significantly further away from the image center than fixations in both Guess conditions (all *p* ≤ 0.003) and fixations in the Count Animals condition were significantly further away from the image center than fixations in the Guess Time condition (*p* = 0.003). Additionally, in the next time interval (800 to 1,200 ms), fixations in the Count Animals condition were significantly further away from the image center than fixations in the Guess Country condition (*p* < .001), but there were still no significant differences both within Guess and within Count conditions (all *p* > 0.8). For the later fixations (1,200 to 8,000 ms), all tasks differed significantly (all *p* ≤ 0.01).

### Entropy

**Figure 9.**
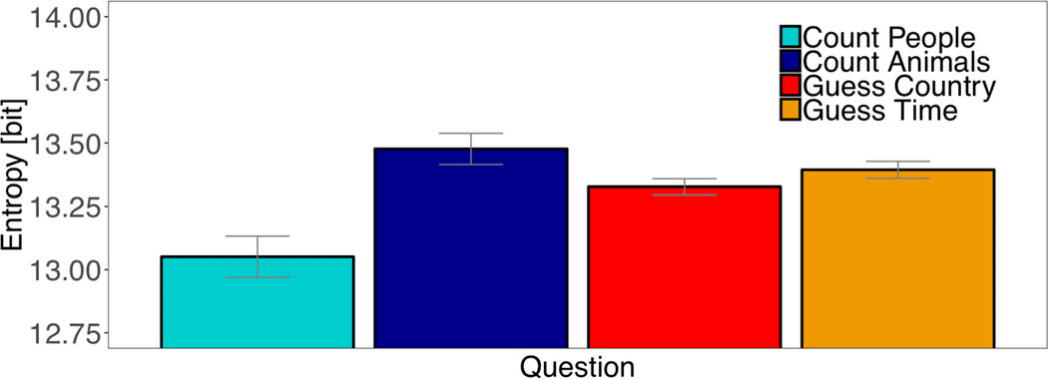
Shannon's entropy. Average entropy of fixation densities on an image in the four tasks. A value of 14 bit is expected for a uniform fixation density. Smaller values indicate that fixations cluster in specific parts of an image. Confidence intervals were corrected for within-subject designs (Cousineau, 2005; Morey, 2008).

We computed Shannon's entropy, Equation (2), as a measure to describe the overall distribution of fixation locations on an image (Figure 9). If all fixations are at the same location, Shannon's entropy would be 0 bit. If all locations are fixated equally often, that is, distributed uniformly, a value of 14 bit would be expected. The entropy of fixation locations in the Count People condition differed the most from a uniform distribution (13.051 bit). The entropy of the Count Animals condition was closest to a uniform distribution (13.476 bit). The values of the entropy of Guess Country (13.327 bit) and Guess Time (13.394 bit) lay between the two Count tasks. A LMM comparing the entropy of the four tasks showed significant differences across all our contrasts. Fixations in Guess conditions are significantly more distributed over the images than fixations in Count conditions (*t* = 2.12; Table 8). Fixations in the Count Animals condition are more widely spread over the images than those from Count People condition (*t* = 3.73) and fixations in the Guess Country task are more distributed than fixation locations measured in the Guess Time task (*t* = 2.06). Post hoc multiple comparison analysis (Table 9) revealed that the Count People condition differed significantly from all other conditions (all *p* ≤ 0.001). There were no other significant differences between tasks (all *p* >.1).

**Table 8.** Fixed effects of linear mixed − effect model (LMM): Entropy for our contrasts. *Note*: |*t*| > 2 are interpreted as significant effects.

	β	*SE*	*t*
Guess - Count	0.09	0.04	2.12
Count Animals - CountPeople	0.39	0.11	3.73
GuessTime - GuessCountry	0.07	0.03	2.06

**Table 9.** Multiple comparisons of entropy for all tasks. Adjusted *p* values reported (Tukey). Levels of significance: ***p < 0.001, **p < 0.01, *p < 0.05.

Post hoc comparison	Estimate	*SE*	*z* value	Pr(>|*z*|)
Count Animals - Count People	0.394	0.105	3.733	<0.001***
Guess Country - Count People	0.255	0.071	3.612	0.001**
Guess Time - Count People	0.324	0.071	4.599	<0.001***
Guess Country - Count Animals	−0.139	0.071	−1.967	0.178
Guess Time - Count Animals	−0.069	0.071	−0.981	0.736
Guess Time - Guess Country	0.070	0.034	2.060	0.147

### Predictability

**Figure 10.**
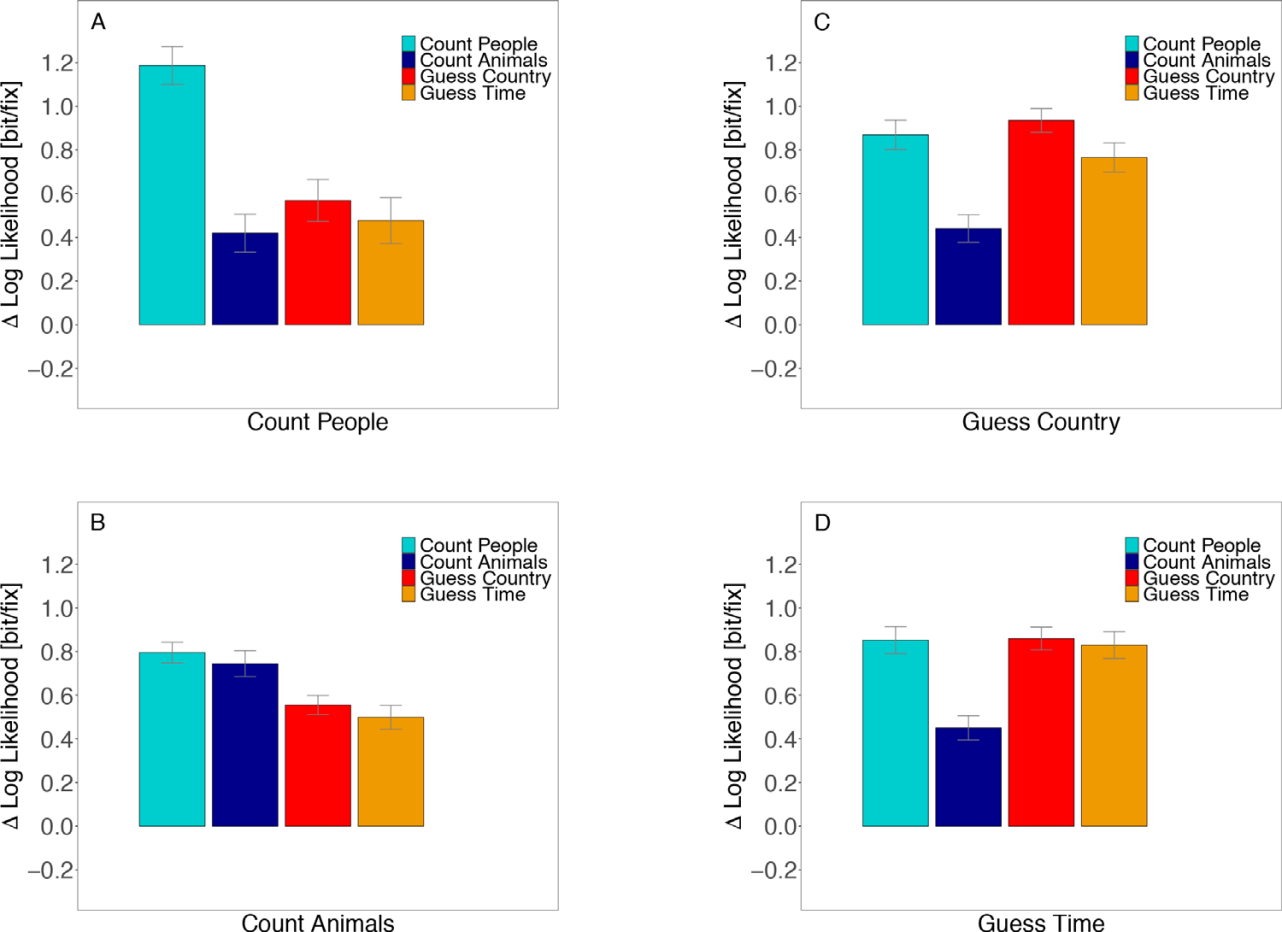
Average predictability of fixation locations in a task. Predictability was measured in bit per fixation as the average gain in log-likelihood of each fixation relative to a uniform distribution. Fixations were predicted from the distribution of all fixation locations measured under (A) Count People, (B) Count Animals, (C) Guess Country, and (D) Guess Time instruction. Confidence intervals were corrected for within-subject designs (Cousineau, 2005; Morey, 2008).

Next, we computed negative cross-entropies of fixation densities to investigate how well fixation locations from one observer viewing an image under a specific instruction can be predicted by the distribution of fixation locations from other observers viewing the same image under one of the four instructions (Figure 10). Panels correspond to how well fixation locations are predicted by the distribution of all other observers viewing an image under the Count People (A), Count Animals (B), Guess Country (C), and Guess Time instruction (D). We report log-likelihood differences, which give the average gain in the log-likelihood per fixation relative to a uniform distribution (Equation 3).

In a first step, we compared how well fixations of one observer viewing an image were on average predicted by other observers viewing the same image under the same instruction. The values correspond to the cyan bar in Panel A, the blue bar in Panel B, the red bar in Panel C, and the orange bar in Panel D (Figure 10). A linear mixed-effect model revealed that both within Guess and within Count conditions differ significantly from each other. Fixations in the Count People condition are more predictable than those in the Count Animals condition (*t* = −4.54; Table 10). And fixations in the Guess Country condition are better to predict than fixations of the Guess Time task (*t* = −2.24). Post hoc multiple comparisons (Table 11) revealed that predictability of fixation locations differed significantly between all tasks (all *p* ≤ 0.025) except for the two Guess conditions (*p* = 0.104) and the Guess Time and Count Animals condition comparison (*p* = 0.209). Thus, when fixations were predicted by other observers viewing an image under the same instruction, fixations from the Count People condition ($1.19\;\frac{\mathrm{bit}}{\text{fix}}$) were better predicted than fixations in the Guess Country ($0.94\;\frac{\mathrm{bit}}{\text{fix}}$) and Guess Time ($0.83\;\frac{\mathrm{bit}}{\text{fix}}$) conditions, which in turn were better predicted than fixations in the Count Animals condition ($0.74\;\frac{\mathrm{bit}}{\text{fix}}$).

**Table 10.** Fixed effects of linear mixed − effect model (LMM): Predictability for our contrasts. *Note*: |*t*| > 2 are interpreted as significant effects.

	β	*SE*	*t*
Guess - Count	−0.03	0.05	−0.62
Count Animals - CountPeople	−0.39	0.09	−4.54
GuessTime - GuessCountry	−0.08	0.04	−2.24

**Table 11.** Multiple comparisons of predictability for all tasks. Adjusted *p* values reported (Tukey). Levels of significance: ***p < 0.001, **p < 0.01, *p < 0.05.

Post hoc comparison	Estimate	*SE*	*z* value	Pr(>|*z*|)
Count Animals - Count People	−0.388	0.086	−4.538	<0.001***
Guess Country - Count People	−0.182	0.066	−2.782	0.025*
Guess Time - Count People	−0.263	0.066	−4.015	<0.001***
Guess Country - Count Animals	0.206	0.065	3.147	0.008**
Guess Time - Count Animals	0.125	0.065	1.913	0.209
Guess Time - Guess Country	−0.081	0.036	−2.240	0.104

In a second step, we investigated whether predictions of the same task differed from the predictions of other tasks. Figure 10A shows how well the distribution of fixation locations from the Count People condition predicted fixation locations of another observer viewing the same image under one of the four instructions. As expected, the distribution of fixation locations from the Count People condition predicted fixation locations in the Count People condition better than fixations in any other condition ($∼1.2\;\frac{\mathrm{bit}}{\text{fix}}$ vs. $∼0.5\;\frac{\mathrm{bit}}{\text{fix}}$). We computed a LMM with treatment contrasts of the fixed factors to test the deviations from the Count People condition. Our analysis confirmed that all conditions differed significantly from the Count People condition (all |*t*| ≥ 20.92; Table 12).

**Table 12.** Fixed effects of linear mixed − effect models (LMM): Predictability with treatment contrasts for the gain in log-likelihood over a uniform distribution. Each block represents the predictions based on the distribution of fixation locations from one task. The intercept corresponds to a prediction of the same task; treatment contrasts represent deviations from this prediction. *Note*: |*t*| > 2 are interpreted as significant effects.

Treatment comparison	β	*SE*	*t*
*Count People*			
Intercept: Count People	1.19	0.07	16.89
Count Animals - Count People	−0.77	0.03	−28.17
Guess Country - Count People	−0.59	0.03	−20.92
Guess Time - Count People	−0.69	0.03	−24.57
*Count Animals*			
Intercept: Count Animals	0.75	0.06	12.74
Count People - Count Animals	0.06	0.02	3.81
Guess Country - Count Animals	−0.19	0.02	−11.83
Guess Time - Count Animals	−0.24	0.02	−14.97
*Guess Country*			
Intercept: Guess Country	0.94	0.07	13.48
Count People - Guess Country	−0.05	0.02	−2.70
Count Animals - Guess Country	−0.50	0.02	−26.16
Guess Time - Guess Country	−0.14	0.02	−7.26
*Guess Time*			
Intercept: Guess Time	0.86	0.06	13.53
Count People - Guess Time	0.02	0.02	1.02
Count Animals - Guess Time	−0.40	0.02	−22.57
Guess Country - Guess Time	0.01	0.02	0.77

Likewise, our analysis confirmed that all conditions differed significantly from the Guess Country condition. Figure 10C shows that the distribution of fixation locations from the Guess Country condition differed significantly in their prediction of fixation locations of another observer viewing the same image under the Guess Country task versus one of the other instructions (all |*t*| ≥ 2.70). While fixation locations were best predicted by the same task in the Count People and the Guess Country conditions, the results for the other conditions were less clear-cut. For the Count Animals condition (Figure 10B), we also found significance across all treatment contrasts (all |*t*| ≥ 3.81), but the distribution of fixation locations from the Count Animals condition predicted fixation locations of other observers viewing the same image under the Count People condition better than fixation locations of other observers viewing the same image under the Count Animals condition (β = 0.06). And finally, predictions of the Guess Time condition (Figure 10D) did not reveal differences between Guess Time, Guess Country, and Count People (all |*t*| ≤  1.02), while predictions of fixation locations in the Count Animals condition were significantly reduced (*t* = −22.57).

### Saliency

**Figure 11.**
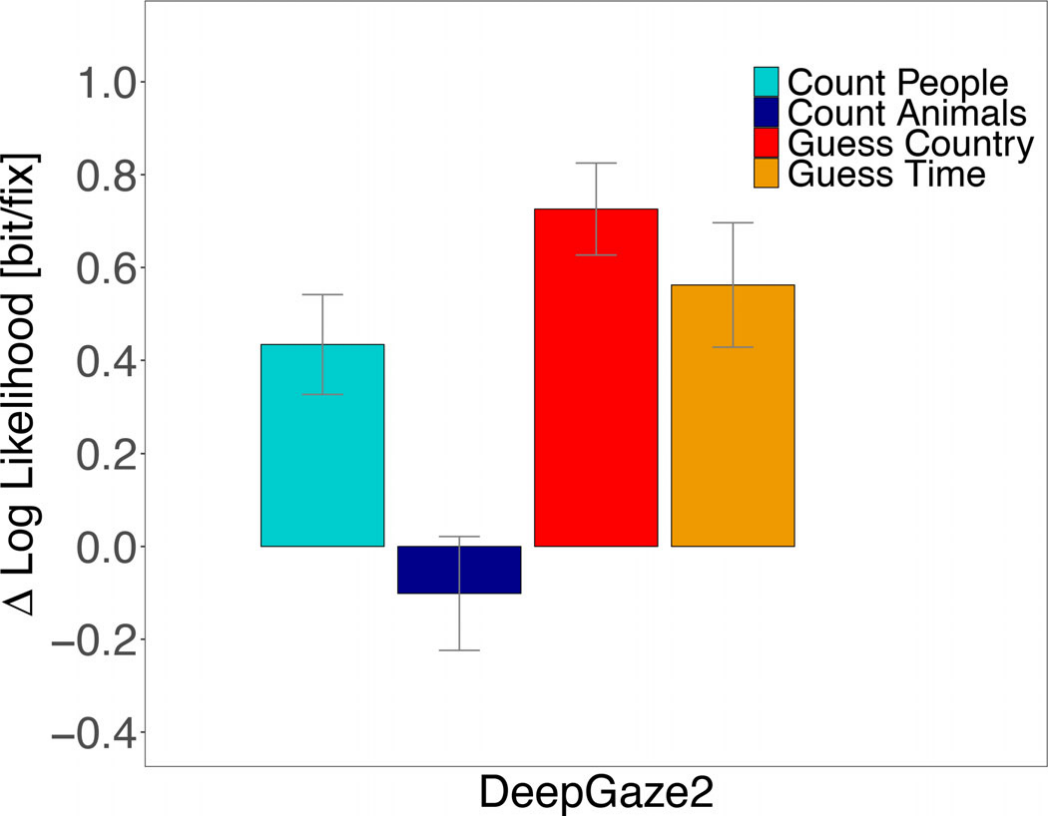
Average predictability of fixation locations in each task by the DeepGaze2 model. Predictability was measured in bit per fixation as the average gain in log-likelihood of each fixation relative to a uniform distribution. Confidence intervals were corrected for within-subject designs (Cousineau, 2005; Morey, 2008).

Finally, we evaluated whether fixation locations in the four tasks can be predicted by the currently most successful saliency model (DeepGaze2; Kümmerer et al., 2016). For each task, we computed the log-likelihood gain of the DeepGaze2 model over a uniform prediction (Figure 11). We chose DeepGaze2 on the basis that it is currently the best-performing saliency model in the MIT-saliency benchmark (Bylinskii et al., 2016) and selected the model option that took the central fixation bias from the MIT1003 data set (Judd et al., 2009) into account. Images were downsampled to 128 × 128 pixels and uploaded to the authors’ web interface deepgaze.bethgelab.org that provided the model predictions. As the predictions are computed in units of natural logarithm, we converted all log-likelihoods to base 2.

Since DeepGaze2 was developed to predict eye movements in scene viewing, our results show that fixation locations in the Guess Country condition were most similar to fixation locations in scene viewing ($∼0.7\;\frac{\mathrm{bit}}{\text{fix}}$). Fixation locations in the Guess Time and Count People conditions were also predicted better than by a uniform distribution ($∼0.5\;\frac{\mathrm{bit}}{\text{fix}}$ and $∼0.4\;\frac{\mathrm{bit}}{\text{fix}}$). In contrast, fixation locations in the Count Animals condition were not well predicted by DeepGaze2. Performance was not better than predictions by a uniform distribution of fixation locations ($∼-0.1\;\frac{\mathrm{bit}}{\text{fix}}$). A linear mixed-effect model revealed significant differences of our three specified contrasts. Fixation locations in Guess conditions can be better predicted by DeepGaze2 than in Count conditions (*t* = 6.11; Table 13). Predictions of fixation locations in the Count People task differed significantly from the Count Animals task (*t* = −4.07) and fixation locations of the Guess Country condition showed better predictability by DeepGaze2 than fixation locations of Guess Time conditions (*t* = −2.16). Post hoc multiple comparisons are listed in Table 14. Predictability of fixation locations differed significantly between all tasks (all *p* < 0.05) except for the Count People and the Guess conditions (all *p* > 0.08).

**Table 13.** Fixed effects of linear mixed − effect model (LMM): DeepGaze2 predictability gain for our contrasts. *Note*: |*t*| > 2 are interpreted as significant effects.

	β	*SE*	*t*
Guess - Count	0.46	0.08	6.11
Count Animals - CountPeople	−0.59	0.15	−4.07
GuessTime - GuessCountry	−0.19	0.07	−2.61

**Table 14.** Multiple comparisons of DeepGaze2 predictability gain for all tasks. Adjusted *p* values reported (Tukey). Levels of significance: ***p < 0.001, **p < 0.01, *p < 0.05.

Post hoc comparison	Estimate	*SE*	*z* value	Pr(>|*z*|)
Count Animals - Count People	−0.591	0.145	−4.066	<0.001***
Guess Country - Count People	0.261	0.111	2.350	0.082
Guess Time - Count People	0.074	0.111	0.666	0.906
Guess Country - Count Animals	0.852	0.111	7.678	<0.001***
Guess Time - Count Animals	0.665	0.111	5.996	<0.001***
Guess Time - Guess Country	−0.187	0.072	−2.609	0.041*

## Discussion

Eye movements during scene viewing are typically studied to investigate the allocation of visual attention on natural, ecologically valid stimuli while keeping the benefits of a highly controlled laboratory setup. However, several aspects of the scene-viewing paradigm have been criticized that question the generalizability of results, and a paradigmatic shift toward the study of natural tasks has been proposed (Tatler et al., 2011). Here, we demonstrate how to adapt the scene-viewing paradigm to make a smooth transition from the scene-viewing paradigm to more natural tasks. This transition allows us to keep the high experimental control of a laboratory setting, bases new research on a solid theoretical ground, and simultaneously deals with the limitations of the classical scene-viewing paradigm.

As a starting point, we demonstrated the general viability of our approach, where we used mobile eye-tracking and a projective transformation to convert gaze coordinates from head-centered coordinates into image-centered coordinates. In the experiment, participants were allowed to move their body and head, since we took away the chinrest, but we did not induce interaction with the stimulus material, which might have produced different gaze patterns (Epelboim et al., 1995). In the presence of such interaction, the control of the gaze deployment system might be rather different. Therefore, we kept interaction at minimum in the current study. However, care has to be taken in follow-up studies that include forms of interaction with stimuli for even more natural behavior. They viewed the same images under four different instructions. We implemented two counting instructions, where participants had to determine the number of people or animals present in a given image. In the two remaining conditions, participants were asked to guess the country, where the given image was taken, or the time of day, at which the image was recorded. Our analyses replicated the sensitivity of various eye-movement measures to specific tasks (Castelhano et al., 2009; DeAngelus & Pelz, 2009; Mills et al., 2011). We observed differences between tasks in fixation durations, saccade amplitudes, strength of the central fixation bias, and eye-movement measures related to distributions of fixation locations. Furthermore, fixation locations in the four tasks were reasonably well predicted by a recent saliency model (Kümmerer et al., 2016).

### Central fixation bias across tasks

An important observation in our study concerned the central fixation bias (Tatler, 2007). While it is well documented that viewers prefer to fixate near the center of images and that this behavior generalizes to other tasks (Ioannidou et al., 2016), a direct within-subject comparison of the central fixation bias across tasks on the same stimulus material has not been reported before. As the central fixation bias typically is strongest during initial fixations (Rothkegel et al., 2017; Tatler, 2007; ’t Hart et al., 2009), we investigated the temporal evolution of the central fixation bias in the four tasks. We observed a strong initial response toward the image center on the earliest fixations and found no differences in the strength of the early central fixation bias between tasks. The central fixation bias decreased on later fixations and reached an asymptotic behavior after 1,000 to 2,000 ms. Interestingly, from the second inspected time interval (400 to 800 ms) onward, the central fixation bias depended on the task given to a participant. Our data suggest a task-independent early central fixation bias and a later task-dependent central fixation bias that reflects differences in the selection of fixation locations during exploration.

### Predictability of fixation locations across tasks

Since their seminal work (Buswell, 1935; Yarbus, 1967), it has been known that eye movements on an image depend on the instruction given to an observer. While task differences have often been replicated (Castelhano et al., 2009; DeAngelus & Pelz, 2009; Mills et al., 2011), prediction of a specific task from a given eye-movement trace has resulted in incoherent success. While Greene et al. (2012) reported a failure to recover task from eye movements reliably, Borji and Itti (2014) demonstrated successful prediction of task from eye movements using the same data set. Here, we investigated how well fixation locations can be predicted by the distribution of fixation locations from other participants viewing an image under the same or a different instruction (Schütt et al., 2019). We made three important observations.

First, when fixation locations were predicted by fixations of other observers viewing an image under the same instruction, predictability of fixation locations differed across tasks. The log-likelihood gain relative to a uniform distribution was highest in the Count People condition, lowest in the Count Animals condition, and in between in the two Guess conditions. Thus, there was no simple relation in predictability between the Count and Guess instructions. The entropy of the fixation location distributions resembled this result. Fixation locations deviated the most from a uniform distribution in the Count People condition and deviated the least from a uniform distribution in the Count Animals condition. Thus, predictability in our tasks can at least partially be explained by the degree of aggregation of fixation locations in the four tasks. It is important to note, however, that this relation is not mandatory, as the entropy only affects the upper limit of the predictability measure. Our results demonstrate that the chosen task influences the interobserver predictability of fixation locations and confirms the need to deliberately choose an instruction in the scene-viewing paradigm that is appropriate for the research question.

Second, we compared predictability of fixation locations across tasks. In general, log-likelihood gains were highest for fixation locations predicted by other participants viewing an image under the same instruction in the majority of tasks. However, fixation location distributions from half of the tasks were not very specific in their predictions, and log-likelihood gains for at least one other task were as high as the log-likelihood gains for the task itself or another log-likelihood gain for another task was higher. Thus, while it is possible to find tasks that lead to very different distributions of fixation locations (Buswell, 1935; Yarbus, 1967), many tasks will result in overlapping distributions, at least on static images in a laboratory setup. The strong overlap in fixation locations between some tasks makes it difficult to differentiate these tasks on the basis of their fixation locations.

Third, fixation locations recorded in the Count People condition showed a distinct pattern. While fixation locations from the Count People condition were well predicted by all other tasks, fixations from the Count People condition primarily predicted fixations from the task itself. We believe that this asymmetry arose from the peculiar role of people and faces for eye movements on images. It is well known that people and faces attract gaze in scene viewing (Cerf et al., 2007; Judd et al., 2009) and that at least some of these fixations are placed involuntarily (Cerf et al., 2009). Torralba et al. (2006) showed that participants who had to count the number of people in a scene used their prior spatial knowledge and directed their fixations toward locations likely to contain people. As a consequence, increased fixation probabilities might be caused by expectations of faces/people rather than the actual existence of corresponding features. This effect might even be enhanced in the Count People task, which puts a particular emphasis on people and locations with high expectations to find people, so it is likely that participants made even more fixations in related regions. This interpretation is supported by the low entropy in the Count People condition, which indicates that fixations clustered more in the Count People task than in any other task. Since people and faces attracted gaze in all tasks and in particular in the Count People condition, all tasks were well able to predict fixation locations in the Count People condition. At the same time, the Count People condition mostly predicted fixations on people and faces in the other conditions. Since these are only a fraction of all fixations in the other conditions, predictability performance of the Count People condition was relatively low for these tasks.

### Search vs. free viewing

Images in our experiment were viewed under four different instructions: two Guess and two Count instructions. The Guess instructions were intended to produce gaze behavior similar to free viewing with fewer task constraints than in the Count instructions that require identification of and search for objects. Contrary to free viewing, however, under Guess instructions, eye behavior across participants was expected to be guided more strongly by the same aspects of the image to solve the tasks (e.g., shadows, daylight, vegetation). In the two Count conditions, participants needed to examine the entire image to detect and count all target objects. Thus, both Count tasks were considered a form of search task as they included a search for target objects in an image.

We compared tasks similar to free viewing (Guess) with tasks similar to search (Count) by quantifying how well fixation locations in the four tasks were predicted by a recent saliency model (DeepGaze2; Kümmerer et al., 2016). Since saliency models were designed to predict fixation locations during free viewing, we expected a better match between the predictions of the saliency model and the two free viewing tasks than the two search tasks (cf. Schütt et al., 2019). Numerically, target selection in the Guess conditions was in better agreement with predictions from the saliency model than in the Count conditions. Statistically, the predictions for the Guess conditions outperformed predictions of the Count Animals condition. The Count People condition lay nearby the Guess conditions and did not differ significantly from these. Since saliency models typically incorporate detectors for persons and faces, a large fraction of fixations on persons and faces can be predicted in the Count People condition (cf. Mackay et al., 2012). In summary, the Guess conditions resembled free viewing more than the Count conditions and, consequently, the Guess conditions generated eye movements similar to the free viewing instruction. It is important to note that the DeepGaze2 model included the central fixation tendency, so that the better prediction of the Guess conditions could be partly explained by the stronger central fixation bias in these conditions.

Low predictive power of saliency models for fixation locations in search tasks has also been reported for the search of artificial targets embedded in scenes (Rothkegel et al., 2019; Schütt et al., 2019) as well as for searching images of real-world scenes for real-world objects (Henderson et al., 2007; Foulsham & Underwood, 2008). While eye-movement parameters like fixation durations and saccade amplitudes adapted to the visibility of the target in the periphery (Rothkegel et al., 2019), fixations were differently associated with features in search and free viewing tasks. Even training a saliency model based on early visual processing to the data set did not improve predictions considerably (Schütt et al., 2019). Our results demonstrate that the low predictive power of saliency models in the search tasks is also true for search tasks with non manipulated real-world scenes. However, while fixation locations were not well predicted by the saliency model in the search tasks and in particular not in the Count Animals tasks, several other eye-movement parameters adapted to the search task. Fixation durations were shortest in the Count Animals condition and saccade amplitudes were shorter and the central fixation bias smaller in the Count conditions than the Guess conditions. Thus, there is no simple relation between low-level image features and fixation locations in search, but other parameters demonstrate that eye movements adapt to the specificities of the task.

### Conclusions

Due to several limitations, the generalizability of theoretical implications of the scene-viewing paradigm has been criticized. However, real-world scenarios often lack experimental control and are detached from the previous research. Here we demonstrate that the advancements in mobile eye-tracking and image processing make it possible to deal with the limitations of the scene-viewing paradigm, while keeping high experimental control in a laboratory setup. Our setup provides a fruitful, highly controlled, but less constrained environment to investigate eye-movement control across tasks.

## Supplementary Material

Supplement 1
