# Supplementary material for: Task-dependence in scene perception: Head unrestrained viewing using mobile eye-tracking
Source: J Vis. 2020 May 11;20(5):3. doi: 10.1167/jov.20.5.3 (PMC7409614; doi:10.1167/jov.20.5.3)
Supplement: Supplement 1 [file jovi-20-5-3_s001.pdf]

# Appendix 1

For our analyses, we used linear mixed effect models (LMM). For each dependent variable, we used the same fixed effect structure, except when explicitly stated otherwise (see Methods for details). For the random effect structure, we estimate random effects for participants and images. We first formulated the maximal possible random effect structure (Barr et al., 2013), with random intercepts for images and participants and random slopes for each of our three contrasts. We reduced these models until the *lme4*-package returned no convergence problems. First, we removed correlation terms. Second, we removed the least varying random effect terms. If the reduced model converges, we try to re-include the correlation terms. In the case that we end up with two converging models of the same complexity, but with different terms for slopes in the image and participant random effect part, we used Bayesian-Information-Criterion (BIC) to determine the best model. We performed a principal component analysis to check whether all random effect terms explain non-zero variance; thus, none of the models was degenerate (Bates et al., 2015). The random effect structure for entropy differed from all others since the entropy measurement is based on fixations from all participants over images, we did not estimate random effects for participants.

Table 1: Random effects structure.

| Dependent variable | | | Random effect participant part | | | Random effect image part | |
|---|---|---|---|---|---|---|---|
| *Fixation duration* | | | | | | | |
| DV∼ | fixed effects | + | (1 + C1 | \|\| participant) | + | (1 + C1 + C2 | \|\| image) |
| *Saccade amplitudes* | | | | | | | |
| DV∼ | fixed effects | + | (1 + C1 | \|\| participant) | + | (1 + C1 + C2 | \|\| image) |
| *Central fixation bias** | | | | | | | |
| DV∼ | fixed effects | + | (1 | \| participant) | + | (1 | \| image) |
| *Entropy* | | | | | | | |
| DV∼ | fixed effects | | | | + | (1 + C1 + C2 | \|\| image) |
| *Predictability* | | | | | | | |
| DV∼ | fixed effects | + | (1 + C1 + C2 | \|\| participant) | + | (1 + C1 + C2 + C3 | \|\| image) |
| *Predictability per task** | | | | | | | |
| DV∼ | fixed effects | + | (1 | \| participant) | + | (1 | \| image) |
| *DeepGaze2* | | | | | | | |
| DV∼ | fixed effects | + | (0 + C1 + C2 + C3 | \|\| participant) | + | (1 + C1 + C2 + C3 | \| image) |

Notes: 1 Intercept, C1 first contrast the two Guess against the two Count tasks, C2 second contrast Count Animals against Count People, C3 third contrast Guess Time against Guess Country, || zero correlation parameter, DV dependent variable, * we choose the minimal model with only random intercepts for participants and images to have comparable models between all subsets of this analysis.

## References


Barr, D. J., Levy, R., Scheepers, C., & Tily, H. J. (2013, April). Random effects structure for confirmatory hypothesis testing: Keep it maximal. *Journal of Memory and Language*, *68*(3), 255–278.

Bates, D., Kliegl, R., Vasishth, S., & Baayen, H. (2015). Parsimonious mixed models. *arXiv preprint arXiv:1506.04967*.
